# Three wave cross-lagged associations between traditional Chinese ethnic sports adherence and anxiety symptoms: parallel mediating roles of emotion regulation difficulties and perceived stress

**DOI:** 10.3389/fpsyg.2026.1904091

**Published:** 2026-09-08

**Authors:** Xuefeng Fan

**Affiliations:** Department of Physical Education, Tianjin Chengjian University, Tianjin, China

**Keywords:** anxiety symptoms, cross-lagged panel model, emotion regulation difficulties, exercise adherence, perceived stress, traditional Chinese ethnic sports

## Abstract

**Background:**

Anxiety symptoms are common among college students. This study examined whether adherence to traditional Chinese ethnic sports was longitudinally associated with anxiety symptoms and whether emotion regulation difficulties and perceived stress served as parallel mediators.

**Methods:**

A three-wave longitudinal survey was conducted among 546 college students from two universities in Tianjin, China. Adherence to traditional Chinese ethnic sports, emotion regulation difficulties, perceived stress, and anxiety symptoms were measured at all three waves. Reliability analysis, common method bias testing, longitudinal measurement invariance, cross-lagged panel modeling, and longitudinal parallel mediation analysis were performed.

**Results:**

All scales showed good internal consistency across waves. The cross-lagged model indicated significant autoregressive effects for all variables. Higher adherence was prospectively associated with lower subsequent emotion regulation difficulties and perceived stress, whereas higher levels of both mediators were prospectively associated with higher later anxiety symptoms. The longitudinal parallel mediation model showed good fit, χ^2^(48) = 71.387, CFI = 0.997, TLI = 0.995, RMSEA = 0.030, and SRMR = 0.017. Bootstrap results supported significant but modest indirect associations through emotion regulation difficulties and perceived stress.

**Conclusion:**

Adherence to traditional Chinese ethnic sports was prospectively associated with lower anxiety symptoms through two modest indirect pathways involving lower emotion regulation difficulties and perceived stress. These observational findings highlight the potential relevance of multidimensional adherence quality for university mental health promotion but do not establish causal effects.

## 1 Introduction

With the global expansion of higher education, mental health problems among university students have become an increasingly prominent public health concern (Auerbach et al., 2018; Li et al., 2022). Anxiety symptoms, in particular, are highly prevalent during emerging adulthood, a developmental period characterized by academic pressure, identity exploration, social adaptation, and uncertainty about future careers (Auerbach et al., 2016; Ahmed et al., 2023). Anxiety not only impairs students’ learning efficiency and quality of life but may also contribute to more serious psychological consequences, including academic withdrawal, interpersonal dysfunction, and long-term maladjustment (Auerbach et al., 2016; Tang et al., 2023). A growing body of epidemiological evidence suggests that anxiety symptoms among college students are substantially more common than in the general population (Li et al., 2022; Ahmed et al., 2023). In China, this issue is similarly concerning, with recent studies indicating that a considerable proportion of college students report clinically relevant anxiety symptoms (Deng et al., 2021; Wang et al., 2022). These findings highlight the urgent need for accessible, sustainable, and culturally appropriate approaches to reducing anxiety and promoting psychological well-being in university settings (Huang et al., 2024; Liu et al., 2025; Li et al., 2025).

Among various non-pharmacological strategies, physical activity has been widely recognized as an effective and low-cost approach for improving mental health (Huang et al., 2024; Liu et al., 2025). Within the Chinese cultural context, traditional Chinese ethnic sports and mind–body exercises, such as Tai Chi, Baduanjin, Yi Jin Jing, shuttlecock kicking, dragon dance, and other culturally embedded movement practices, may offer unique psychological benefits (Lin et al., 2022; Qi et al., 2022; Dong et al., 2023). These activities typically integrate bodily movement, breath regulation, attentional focus, rhythm, coordination, and cultural meaning (Wayne and Kaptchuk, 2008; Qi et al., 2022). Compared with conventional physical exercise, traditional Chinese ethnic sports are not merely forms of bodily training; they also involve embodied cultural experience, self-cultivation, social interaction, and emotional balance (Wayne and Kaptchuk, 2008; He et al., 2025). Previous intervention studies and systematic reviews have shown that traditional Chinese mind–body exercises can effectively alleviate anxiety and depressive symptoms among college students (Du et al., 2022; Lin et al., 2022; Qi et al., 2022). Such evidence suggests that traditional Chinese ethnic sports may serve as a culturally meaningful pathway for mental health promotion on university campuses (Du et al., 2022; Lin et al., 2022).

However, most existing studies have focused primarily on whether students participate in traditional exercise, how often they participate, or how long they exercise (Huang et al., 2024; He et al., 2025). The present study conceptualizes exercise adherence as a multidimensional implementation construct rather than as attendance or participation alone. Specifically, adherence reflects cognitive understanding of the exercise prescription, behavioral enactment of prescribed frequency, intensity, time, type, volume, and progression, and self-regulatory capacity to maintain and adjust practice (Rhodes and Fiala, 2009; Garber et al., 2011; He et al., 2025). Participation therefore indicates exposure to an activity, whereas adherence indicates the extent to which the intended program is actually implemented. This distinction is theoretically important because the psychological features of traditional sports, including repeated attentional focus, breath regulation, movement control, and progressive practice, can only be experienced consistently when the prescription is enacted with sufficient quality and continuity.

This issue is particularly relevant in Chinese university physical education contexts. Many college students encounter traditional Chinese ethnic sports through compulsory or elective physical education courses (Qi et al., 2022; He et al., 2025), but course enrollment does not guarantee that they understand, implement, and self-regulate the prescribed practice. Some students may attend passively, practice irregularly, or fail to meet the intended standards of frequency, intensity, or progression (Garber et al., 2011; He et al., 2025). A critical research gap is therefore whether students’ overall multidimensional adherence, rather than mere participation, is prospectively associated with lower subsequent anxiety symptoms (Cole and Maxwell, 2003; Maxwell and Cole, 2007; He et al., 2025). Addressing this gap shifts the question from whether traditional ethnic sports are generally beneficial to whether their intended active components are implemented consistently and through which explanatory pathways adherence is associated with later psychological adjustment (Cole and Maxwell, 2003; Maxwell and Cole, 2007).

In addition to examining the direct longitudinal association between adherence and anxiety symptoms, it is necessary to clarify potential explanatory pathways underlying this relationship (Cole and Maxwell, 2003; Sheng et al., 2024). Two constructs are especially relevant: emotion regulation difficulties and perceived stress (Cohen et al., 1983; Gratz and Roemer, 2004). Emotion regulation difficulties refer to deficits in awareness, understanding, acceptance, and modulation of emotional responses, as well as difficulties maintaining goal-directed behavior and controlling impulses under distress (Gratz and Roemer, 2004). Emotion regulation theory proposes that individuals who have difficulty identifying and managing negative emotional states are more likely to experience persistent arousal, maladaptive coping, and vulnerability to internalizing symptoms (Gross, 1998; Sloan et al., 2017). Among college students, these difficulties may appear as poor emotional awareness, limited access to regulation strategies, impulsive reactions, and disrupted academic or interpersonal functioning under negative affect (Gratz and Roemer, 2004; Sheng et al., 2024).

Traditional Chinese ethnic sports may support more adaptive emotion regulation through repeated embodied practice (Wayne and Kaptchuk, 2008; Sheng et al., 2024). These activities often require coordination of movement and breathing, sustained attention, modulation of bodily tension, and cultivation of a calm internal state (Wayne and Kaptchuk, 2008; Qi et al., 2022). Such practice may provide repeated opportunities to monitor internal experiences, tolerate discomfort, and regulate physiological arousal in a structured and culturally meaningful context (Gross, 1998; Wayne and Kaptchuk, 2008). Accordingly, higher overall adherence may be prospectively associated with fewer later emotion regulation difficulties because students more consistently encounter these regulatory demands and practice opportunities (He et al., 2025; Sheng et al., 2024). Emotion regulation difficulties are therefore examined as one potential indirect pathway linking adherence with later anxiety symptoms (Gratz and Roemer, 2004; Sloan et al., 2017).

Perceived stress represents a second potential explanatory pathway. According to stress appraisal theory, stress is shaped not only by external demands but also by the appraisal that those demands exceed available coping resources (Folkman et al., 1986; Lazarus, 1993). University students frequently encounter academic workload, interpersonal challenges, financial concerns, and career uncertainty (Wunsch et al., 2021; Zhao et al., 2023). Higher perceived stress is associated with worry, physiological arousal, threat-focused attention, and avoidance, all of which are closely related to anxiety symptoms (Cohen et al., 1983; Zhao et al., 2023). Prior research has linked physical activity with lower perceived stress, possibly through stronger coping resources, self-efficacy, and physiological recovery (VanKim and Nelson, 2013; Wunsch et al., 2021). However, it remains insufficiently understood whether overall adherence to traditional Chinese ethnic sports is prospectively associated with later anxiety symptoms through lower perceived stress (He et al., 2025; Wang et al., 2025).

Emotion regulation difficulties and perceived stress may operate as distinct but complementary explanatory pathways. Emotion regulation difficulties capture deficits in awareness, acceptance, and modulation of emotional responses, whereas perceived stress reflects the appraisal that environmental demands exceed available coping resources (Cohen et al., 1983; Gratz and Roemer, 2004). Adherence to traditional Chinese ethnic sports may be prospectively associated with lower anxiety through either pathway: repeated embodied practice may support emotional self-regulation, while structured and culturally meaningful exercise may be associated with lower perceived uncontrollability and overload of daily demands (Gross, 1998; Folkman et al., 1986; Wayne and Kaptchuk, 2008). Because both mediators were assessed at Time 2, the present study did not impose a causal ordering between them. Instead, emotion regulation difficulties and perceived stress were specified as parallel mediators, allowing each indirect pathway to be estimated while accounting for the other mediator.

Several limitations remain in the current literature. First, most studies on traditional Chinese exercise and college students’ mental health have relied on cross-sectional or short-term intervention designs, limiting the ability to establish temporal precedence among variables (Cole and Maxwell, 2003; Maxwell and Cole, 2007). Cross-sectional mediation models are particularly insufficient for testing developmental or psychological processes because the presumed cause, mediator, and outcome are measured simultaneously (Maxwell and Cole, 2007). Second, previous studies have tended to examine traditional exercise participation rather than adherence quality, leaving unclear whether students who follow exercise prescriptions more consistently experience greater psychological benefits (He et al., 2025). Third, although emotion regulation difficulties and perceived stress have each been linked to anxiety, few studies have examined them simultaneously as parallel mediators in the association between traditional Chinese ethnic sports adherence and later anxiety symptoms (Sheng et al., 2024; Wang et al., 2025). These gaps suggest the need for a longitudinal design that can better capture the temporal ordering of overall multidimensional adherence, parallel explanatory pathways, and later anxiety outcomes (Cole and Maxwell, 2003; Maxwell and Cole, 2007).

To address these limitations, the present study employed a three-wave longitudinal design to examine associations between adherence to traditional Chinese ethnic sports and anxiety symptoms among college students (Cole and Maxwell, 2003; Maxwell and Cole, 2007). All four constructs were measured at every wave. The focal mediation analysis used overall adherence at Time 1, emotion regulation difficulties and perceived stress at Time 2, and anxiety symptoms at Time 3, while controlling baseline levels of the mediators and outcome. The remaining repeated measurements were used to establish longitudinal measurement invariance and to estimate reciprocal cross-lagged associations. This specification temporally separates the antecedent, mediators, and outcome, but it does not represent a fully dynamic mediation model incorporating every possible wave-specific indirect pathway. By distinguishing multidimensional adherence from simple participation, the study provides a more precise behavioral account of how the implementation quality of traditional Chinese ethnic sports is prospectively associated with mental health (He et al., 2025).

The present study makes several contributions to the literature. First, it shifts the research focus from participation dosage to adherence quality, thereby extending current understanding of the conditions under which traditional Chinese ethnic sports may benefit college students’ mental health (He et al., 2025). Second, it integrates emotion regulation theory and stress appraisal theory to explain how adherence may be prospectively associated with anxiety symptoms (Gross, 1998; Folkman et al., 1986). Third, by using a three-wave longitudinal parallel mediation model, this study moves beyond cross-sectional mediation analysis and provides temporally separated evidence regarding the prospective ordering of overall multidimensional adherence, the two mediators, and anxiety symptoms without assuming a serial relationship between the mediators (Cole and Maxwell, 2003; Maxwell and Cole, 2007). Finally, the findings may offer practical implications for university physical education programs by suggesting that traditional ethnic sports courses should not only increase participation but also foster sustained, high-quality adherence to exercise prescriptions (Garber et al., 2011; He et al., 2025).

The theoretical contribution should therefore be understood as integrative and specification-oriented rather than as a claim that each component association is unprecedented. The study positions multidimensional adherence as the implementation quality of a traditional sports prescription: participation indicates whether exposure occurred, whereas adherence indicates whether cognitive understanding, behavioral execution, and self-regulatory maintenance allowed the intended practice elements to be repeatedly enacted (Rhodes and Fiala, 2009; Garber et al., 2011; He et al., 2025). It also distinguishes two complementary explanatory domains—emotion-regulatory capacity and stress appraisal within the same longitudinal framework (Gross, 1998; Folkman et al., 1986). This framework may help explain why students with similar participation opportunities nevertheless show different psychological outcomes when the quality and sustainability of their practice differ.

Based on the above theoretical and empirical considerations, the following hypotheses were proposed:

*H1*: Higher overall adherence to traditional Chinese ethnic sports at Time 1 will be prospectively associated with lower anxiety symptoms at Time 3.

*H2*: Higher overall adherence at Time 1 will be prospectively associated with lower emotion regulation difficulties at Time 2, which in turn will be associated with lower anxiety symptoms at Time 3.

*H3*: Higher overall adherence at Time 1 will be prospectively associated with lower perceived stress at Time 2, which in turn will be associated with lower anxiety symptoms at Time 3.

*H4*: Emotion regulation difficulties and perceived stress at Time 2 will jointly serve as parallel mediators of the prospective association between overall adherence at Time 1 and anxiety symptoms at Time 3; specifically, the combined indirect effect through the two parallel pathways will be statistically significant.

Taken together, these hypotheses outline a longitudinal explanatory framework in which adherence to traditional Chinese ethnic sports is expected to serve as a prospective behavioral correlate of lower anxiety symptoms among college students (He et al., 2025; Sheng et al., 2024). Rather than assuming that participation alone is sufficient to produce psychological benefits, the present study emphasizes sustained and high-quality adherence to prescribed exercise practices (Garber et al., 2011; Rhodes and Fiala, 2009). By examining emotion regulation difficulties and perceived stress as parallel explanatory pathways, the study tests two theoretically distinct but complementary explanatory pathways through which adherence may be associated with lower anxiety symptoms over time (Cole and Maxwell, 2003). Because both mediators were measured at Time 2, no temporal sequence between emotion regulation difficulties and perceived stress was hypothesized or estimated. The hypotheses concern longitudinal associations and model-based indirect effects rather than experimentally established causal effects.

## 2 Materials and methods

### 2.1 Participants

Participants were recruited from two universities in Tianjin, China using a multistage cluster sampling procedure with purposive institutional selection. At the first stage, two universities offering established traditional Chinese ethnic sports courses were selected to ensure access to the target population. At the second stage, eligible physical education course sections were randomly selected from the course rosters at each university, and all students in the selected classes who met the eligibility criteria were invited to participate. University, academic major or field of study, ethnicity, urban or rural residence, and self-rated family socioeconomic status (low, middle, or high relative to peers) were recorded and are reported in Table 1.

**Table 1:** Sociodemographic characteristics of the sample (*N* = 546).

Variable	Category	*n*	%
Gender	Female	287	52.6
Gender	Male	259	47.4
Grade	Junior	128	23.4
Grade	Freshman	161	29.5
Grade	Senior	80	14.7
Grade	Sophomore	177	32.4
Age	Mean ± SD	20.09 ± 1.23	
Age	Range	18–23	
University	University A	286	52.4
University	University B	260	47.6
Academic major/field	Engineering and technology	181	33.2
Academic major/field	Health and medical sciences	117	21.4
Academic major/field	Humanities and social sciences	104	19.0
Academic major/field	Economics and management	88	16.1
Academic major/field	Arts and education	56	10.3
Ethnicity	Han Chinese	519	95.1
Ethnicity	Ethnic minority / other	27	4.9
Residence	Urban	294	53.8
Residence	Rural	252	46.2
Socioeconomic status	Low	112	20.5
Socioeconomic status	Middle	361	66.1
Socioeconomic status	High	73	13.4

*N* = 546. SD, standard deviation; SES, socioeconomic status. Percentages may differ slightly from 100% because of rounding. ADH, adherence to traditional Chinese ethnic sports (23 items; total score 23–115; item-mean score 1–5); DERS, Difficulties in Emotion Regulation Scale (36 items; total score 36–180; item-mean score 1–5); PSS, 10-item Perceived Stress Scale (10 items; total score 0–40; item-mean score 0–4); GAD/GAD-7, 7-item Generalized Anxiety Disorder scale (7 items; total score 0–21; item-mean score 0–3); T1, T2, and T3, Time 1, Time 2, and Time 3.

An *a priori* sample-size calculation was conducted using G*Power 3.1. Because the focal longitudinal mediation model could be expressed as a set of regression equations, the most demanding regression equation was used for the calculation. Assuming a small effect size of f^2^ = 0.03, *α* = 0.05, statistical power of 0.80, and eight predictors, the minimum required sample was 509 participants. After allowing for an anticipated attrition rate of 20%, the target baseline sample was 637. A total of 636 valid participants were enrolled at baseline, which was essentially consistent with the prespecified target.

The final analytic sample consisted of 546 full-time undergraduate students who completed valid questionnaires across all three measurement waves. All participants were enrolled at one of the two selected universities and had experience participating in traditional Chinese ethnic sports courses or related physical activities, including Tai Chi, Baduanjin, Wushu, shuttlecock kicking, dragon dance, or other traditional ethnic sports activities. Eligibility criteria were: (1) full-time undergraduate enrollment; (2) prior or current participation in a traditional Chinese ethnic sports course or related activity; (3) ability to read and independently complete the questionnaire; and (4) voluntary agreement to participate in the three-wave survey. Students were excluded if they self-reported a physician-diagnosed chronic medical condition that substantially restricted physical activity or a severe psychiatric disorder that impaired informed consent or independent questionnaire completion. These criteria were assessed using a brief self-report screening form at T1; university medical records were not accessed. Questionnaires were excluded when they contained substantial missing data, highly repetitive or patterned responses, failed attention-check items, logically inconsistent responses, or identification codes that could not be matched across waves.

Only participants with valid matched responses at Time 1, Time 2, and Time 3 were included in the final analyses. At T1, 680 questionnaires were distributed and 657 were returned, yielding a response rate of 96.6%. Twenty-one questionnaires were excluded because eight respondents did not meet the eligibility criteria, seven had substantial missing data, and six showed patterned responses or failed attention checks, leaving 636 valid baseline participants. At T2, 594 participants completed the survey (retention = 93.4%; dropout = 6.6%); losses were attributable to absence or inability to contact (*n* = 26), voluntary withdrawal (*n* = 10), and academic leave or transfer (*n* = 6). At T3, 558 participants completed the survey (retention from baseline = 87.7%; cumulative dropout = 12.3%); the additional losses reflected absence or inability to contact (*n* = 22), voluntary withdrawal (*n* = 8), and academic leave or transfer (*n* = 6). Following longitudinal matching and final quality screening, seven cases with unmatched identification codes and five cases with incomplete or invalid responses were excluded. The final matched analytic sample was therefore 546, corresponding to an analytic retention rate of 85.8% from baseline. The sociodemographic characteristics of the retained sample are reported in Table 1.

Attrition analyses compared the 546 participants retained in the final analytic sample with the 90 baseline participants who were not included at T3. The two groups did not differ significantly in age, t(634) = 0.84, *p* = 0.401, d = 0.09; gender, χ^2^(1) = 0.18, *p* = 0.670, Cramér’s V = 0.02; university, χ^2^(1) = 0.52, *p* = 0.472, Cramér’s V = 0.03; academic field, χ^2^(4) = 2.91, *p* = 0.573, Cramér’s V = 0.07; ethnicity, χ^2^(1) = 0.09, *p* = 0.769, Cramér’s V = 0.01; residence, χ^2^(1) = 0.66, *p* = 0.416, Cramér’s V = 0.03; or socioeconomic status, χ^2^(2) = 1.74, *p* = 0.419, Cramér’s V = 0.05. No significant baseline differences were found for ADH, t(634) = −0.73, *p* = 0.466, d = −0.08; DERS, t(634) = 1.03, *p* = 0.303, d = 0.11; PSS, t(634) = 1.21, *p* = 0.228, d = 0.13; or GAD-7, t(634) = 1.36, *p* = 0.175, d = 0.15. All effect sizes were small, suggesting limited evidence of systematic attrition bias.

All participants provided informed consent before data collection. They were informed that participation was voluntary, that their responses would be used only for academic research, and that they could withdraw from the study at any time without penalty. All data were collected anonymously and treated confidentially. The study was conducted in accordance with the ethical principles of the Declaration of Helsinki. Ethical approval was obtained from the Ethics Committee of Tianjin Chengjian University (Approve No: 2024022).

### 2.2 Procedure

This study employed a three-wave longitudinal design conducted from September 2024 to June 2025, with a total duration of approximately 9 months. Time 1 was administered in September 2024 at the beginning of the autumn semester, Time 2 was administered in January 2025 approximately 4 months later, and Time 3 was administered in June 2025 approximately 5 months after Time 2. The same four core constructs were assessed at every wave: adherence to traditional Chinese ethnic sports, emotion regulation difficulties, perceived stress, and anxiety symptoms.

At each measurement wave, participants completed the same set of standardized self-report questionnaires. Repeated assessment of all four variables allowed the study to evaluate the temporal stability of each construct, examine prospective cross-lagged associations among variables, and test whether emotion regulation difficulties and perceived stress served as longitudinal parallel mediators in the association between adherence to traditional Chinese ethnic sports and later anxiety symptoms.

Before data collection, trained members of the research team provided participants with standardized instructions. They explained the purpose of the study, the general survey procedures, the voluntary nature of participation, and the principles of confidentiality and anonymity. Participants were informed that there were no right or wrong answers and were encouraged to respond honestly according to their actual experiences. To reduce response bias, questionnaires were completed independently, and participants were assured that their responses would not affect their academic evaluation or course performance.

Questionnaires were administered either in classroom settings or through a secure online survey platform under the supervision of the research team. For classroom-based surveys, participants completed the questionnaires in a quiet environment with sufficient time provided. For online surveys, participants accessed the questionnaire through a secure link and completed it independently. The same questionnaire content and response format were used across the three waves to ensure consistency in measurement.

To match responses across the three waves while protecting participant anonymity, each participant generated a unique identification code based on non-sensitive personal information. This code was used only for longitudinal data matching and was not used to identify individual participants. No names, student identification numbers, telephone numbers, or other personally identifiable information were included in the analytic dataset.

After each wave of data collection, questionnaires were screened for completeness and response quality. Responses with substantial missing data, invalid identification codes, highly repetitive response patterns, failed attention-check items, or logically inconsistent answers were excluded according to predefined data-screening criteria. Only participants with valid matched responses across Time 1, Time 2, and Time 3 were retained for the final longitudinal analyses.

The three-wave design served two main analytic purposes. First, it enabled a cross-lagged panel model to examine prospective reciprocal associations after accounting for autoregressive stability. Second, it enabled a temporally separated parallel mediation model in which Time 1 adherence was the antecedent, Time 2 emotion regulation difficulties and perceived stress were parallel mediators, and Time 3 anxiety symptoms were the outcome. These analyses were designed to characterize temporal associations and indirect pathways; they were not interpreted as establishing causal effects.

### 2.3 Instruments

All instruments were administered at Time 1 (T1), Time 2 (T2), and Time 3 (T3). The study variables were named consistently as adherence to traditional Chinese ethnic sports (ADH), difficulties in emotion regulation (DERS), perceived stress (PSS), and anxiety symptoms assessed with the Generalized Anxiety Disorder-7 scale (GAD-7). Item-mean composite scores were used in correlation and structural equation analyses so that the analyzed ranges were 1–5 for ADH, 1–5 for DERS, 0–4 for PSS, and 0–3 for GAD-7. Total scores were additionally reported for descriptive interpretation. Reverse-coded items were recoded before total and mean scores were calculated; higher scores consistently represented higher levels of the named construct.

#### 2.3.1 Adherence to traditional Chinese ethnic sports

Adherence to traditional Chinese ethnic sports was assessed using the 23-item Ethnic Traditional Sports Exercise Prescription Adherence Scale developed by He et al. (2025). In this manuscript, the scale score is consistently abbreviated as ADH. The scale evaluates the extent to which individuals adhere to ethnic traditional sports exercise prescriptions and is grounded in the FITT-VP principles of frequency, intensity, time, type, volume, and progression. It covers cognitive adherence, behavioral adherence, and self-regulatory adherence. Accordingly, ADH in all subsequent sections refers to the overall composite of cognitive, behavioral, and self-regulatory adherence; it does not refer to the behavioral subscale alone.

In the present study, this scale was used to assess students’ overall adherence to traditional Chinese ethnic sports practice. Representative item content includes students’ understanding of traditional sports exercise prescriptions, their ability to follow prescribed practice requirements, and their capacity to adjust and maintain practice according to exercise goals. Example items may include statements reflecting whether participants can follow the prescribed frequency of traditional sports practice, maintain appropriate exercise intensity, and adjust their practice plan according to progression requirements.

Participants responded to each item using a 5-point Likert scale, ranging from 1 to 5. Higher scores indicated higher levels of adherence to traditional Chinese ethnic sports exercise prescriptions. The total score was obtained by summing the 23 items, with a possible total score range of 23 to 115. The mean score was calculated by dividing the total score by the number of items, with a possible range of 1 to 5. In the present study, higher ADH scores represented greater adherence to traditional Chinese ethnic sports. The Cronbach’s alpha coefficients for this scale were 0.959 at Time 1, 0.957 at Time 2, and 0.958 at Time 3.

#### 2.3.2 Emotion regulation difficulties

Emotion regulation difficulties were measured using the 36-item Difficulties in Emotion Regulation Scale developed by Gratz and Roemer (2004). The score is consistently abbreviated as DERS and assesses difficulties in emotional awareness, emotional clarity, acceptance of emotional responses, impulse control, access to effective regulation strategies, and goal-directed behavior under emotional distress.

In this study, the DERS was used to assess maladaptive aspects of emotion regulation among college students. Representative item content includes difficulty understanding emotional states, difficulty controlling behavior when upset, difficulty concentrating under emotional distress, and limited access to effective strategies for regulating negative emotions. Example items may include statements reflecting whether participants feel confused about their emotions, have difficulty controlling their behavior when distressed, or find it difficult to continue goal-directed activities when experiencing negative emotions.

Participants rated each item on a 5-point Likert scale, ranging from 1 to 5. Higher scores indicated greater emotion regulation difficulties rather than better emotion regulation ability. After reverse-coded items were recoded according to the scale instructions, item scores were summed to obtain a total score. The possible total score range was 36 to 180, with higher scores representing more severe difficulties in emotion regulation. Mean scores were also calculated for descriptive and longitudinal analyses. In the present study, the Cronbach’s alpha coefficients for the DERS were 0.969 at Time 1, 0.969 at Time 2, and 0.969 at Time 3.

#### 2.3.3 Perceived stress

Perceived stress was assessed using the 10-item Perceived Stress Scale developed by Cohen (Cohen et al., 1983). The PSS measures the degree to which individuals appraise their life circumstances as unpredictable, uncontrollable, and overloaded. It is widely used to assess subjective stress appraisal in nonclinical and student populations.

In the present study, the PSS was used to assess students’ perceived stress during the recent period. Representative item content includes feelings of being unable to control important things in life, feeling nervous or stressed, perceiving difficulties as accumulating, and confidence in handling personal problems. Example items may include statements reflecting whether participants felt unable to control important life events or felt that difficulties were piling up beyond their ability to cope.

Participants responded to each PSS item using a 5-point scale ranging from 0 to 4. Positively worded items were reverse coded before scoring. The total score was obtained by summing all 10 items (possible range: 0–40), and the item-mean score ranged from 0 to 4. Higher PSS scores indicated higher perceived stress. The Cronbach’s alpha coefficients were 0.906 at T1, 0.910 at T2, and 0.906 at T3.

#### 2.3.4 Anxiety symptoms (GAD-7)

Anxiety symptoms were assessed using the 7-item Generalized Anxiety Disorder scale (GAD-7; Spitzer et al., 2006). The construct is referred to consistently as anxiety symptoms, and the scale abbreviation GAD-7 is used when describing measurement. The instrument assesses the frequency and severity of anxiety-related symptoms and has been widely used in clinical, community, and university student samples.

In this study, the GAD was used to measure anxiety symptoms among college students across the three waves. Representative item content includes nervousness, uncontrollable worry, excessive worrying, difficulty relaxing, restlessness, irritability, and fear that something awful might happen. Example items may include statements asking how often participants felt nervous or anxious, were unable to stop worrying, or had difficulty relaxing during the recent period.

Participants rated each GAD-7 item on a 4-point scale ranging from 0 to 3. The total score was calculated by summing the seven items (possible range: 0–21), and the item-mean score ranged from 0 to 3. Higher scores indicated more severe anxiety symptoms. The Cronbach’s alpha coefficients were 0.877 at T1, 0.889 at T2, and 0.895 at T3.

### 2.4 Data analysis

Data analyses were conducted using SPSS and structural equation modeling software AMOS. The analysis proceeded in several steps.

First, data screening was performed. Missing data, outliers, normality, and response quality were examined before the main analyses. Participants with invalid response patterns, excessive missing values, or unmatched longitudinal identification codes were excluded. For valid cases with a small amount of missing data, full information maximum likelihood estimation or multiple imputation was used, depending on the missing data pattern. Descriptive statistics, including means, standard deviations, skewness, and kurtosis, were calculated for all study variables at each time point.

Attrition was evaluated by comparing participants retained in the final three-wave sample with those not included in the final analysis on baseline sociodemographic characteristics and all four study variables. Continuous variables were compared using independent-samples t tests, with Cohen’s d reported as the effect-size index, and categorical variables were compared using chi-square tests, with Cramér’s V reported. The results of these analyses are presented in Section 3.1.

Common method variance was evaluated using Harman’s single-factor test and full-collinearity VIFs as complementary diagnostics. These procedures were not treated as conclusive tests that can rule out method variance, because Harman’s test has limited sensitivity and VIF diagnostics address only particular manifestations of shared-method inflation (Podsakoff et al., 2003; Podsakoff et al., 2012; Kock, 2015). Procedural precautions included anonymous participation, standardized instructions, independent questionnaire completion, neutral wording, and assurances that responses would not affect academic evaluation. The diagnostic findings were therefore interpreted cautiously as showing no dominant single-factor or severe full-collinearity pattern, rather than proving the absence of common method bias.

Second, reliability analyses were conducted for each scale at Time 1, Time 2, and Time 3. Internal consistency was evaluated using Cronbach’s alpha coefficients. Composite reliability may also be reported if structural equation modeling is used. Values above 0.70 were considered acceptable.

Third, Pearson correlation analyses were performed to examine the cross-sectional and longitudinal associations among adherence to traditional Chinese ethnic sports, emotion regulation difficulties, perceived stress, and anxiety symptoms. Correlations among the same variables across the three time points were used to evaluate temporal stability, whereas correlations among different variables provided preliminary evidence for the hypothesized longitudinal relationships.

Fourth, confirmatory factor analysis was conducted to examine the measurement model. The factor structure of each scale was first tested separately, and then a combined measurement model including the four constructs was examined. Model fit was evaluated using commonly recommended fit indices, including the chi-square/degrees of freedom ratio, comparative fit index, Tucker–Lewis index, root mean square error of approximation, and standardized root mean square residual. Values of CFI and TLI greater than 0.90 and values of RMSEA and SRMR lower than 0.08 were considered indicative of acceptable model fit.

Fifth, longitudinal measurement invariance was tested before conducting cross-lagged and longitudinal mediation analyses. Because all four variables were measured repeatedly across three time points, it was necessary to ensure that each construct had the same measurement meaning over time. Measurement invariance was examined sequentially at four levels: configural invariance, metric invariance, scalar invariance, and residual invariance.

Configural invariance was tested to determine whether the same factor structure held across Time 1, Time 2, and Time 3. Metric invariance was tested by constraining factor loadings to be equal across the three time points. Scalar invariance was tested by further constraining item intercepts to equality across time. Residual invariance was tested by constraining item residuals to equality across time. Changes in model fit were evaluated using differences in CFI, RMSEA, and SRMR. Measurement invariance was considered acceptable when the decrease in CFI was less than or equal to 0.010 and the increase in RMSEA was less than or equal to 0.015. Establishing at least metric and scalar invariance was considered necessary for meaningful comparison of longitudinal associations and latent means across time.

Sixth, a three-wave cross-lagged panel model was constructed to examine the reciprocal longitudinal relationships among adherence to traditional Chinese ethnic sports, emotion regulation difficulties, perceived stress, and anxiety symptoms. The model included autoregressive paths for each variable from Time 1 to Time 2 and from Time 2 to Time 3. These autoregressive paths controlled for the temporal stability of each construct. The model also included cross-lagged paths among the four variables across adjacent time points. Within-time correlations among the variables were freely estimated at each wave.

The cross-lagged panel model was used to test whether earlier levels of each construct were prospectively associated with later levels of the other constructs after controlling for autoregressive stability. Because conventional CLPM coefficients can combine stable between-person differences with within-person fluctuations, the coefficients were interpreted as prospective panel associations in relative standing rather than as within-person or causal effects.

A conventional CLPM was retained because the prespecified objective was to examine reciprocal prospective associations among four repeatedly measured constructs at the observed panel level and to maintain a parsimonious model aligned with the focal mediation analysis. Random-intercept cross-lagged panel models explicitly separate stable between-person differences from within-person deviations and are preferable when the primary estimand is an intraindividual process (Hamaker et al., 2015; Mulder and Hamaker, 2021). The present analysis did not target that estimand; consequently, the selected CLPM and its interpretive limits are stated explicitly.

Seventh, a longitudinal parallel mediation model was tested. Overall adherence to traditional Chinese ethnic sports at Time 1 was specified as the antecedent variable, emotion regulation difficulties and perceived stress at Time 2 were specified as parallel mediators, and anxiety symptoms at Time 3 were specified as the outcome. The focal model followed the temporal order of the three-wave design:

T1 adherence to traditional Chinese ethnic sports → T2 emotion regulation difficulties → T3 anxiety symptoms.

T1 adherence to traditional Chinese ethnic sports → T2 perceived stress → T3 anxiety symptoms.

Baseline levels of the mediators and outcome were controlled to strengthen temporal separation. Specifically, Time 1 emotion regulation difficulties, Time 1 perceived stress, and Time 1 anxiety symptoms were included as covariates. The focal paths therefore estimated prospective associations of Time 1 adherence with Time 2 mediator levels, and of Time 2 mediator levels with Time 3 anxiety symptoms, conditional on the corresponding baseline scores; they should not be interpreted as causal changes.

Although all four constructs were available at all three waves, the focal indirect model intentionally used the prespecified Time 1 → Time 2 → Time 3 contrast. Time 2 adherence and Time 3 mediator scores contributed to the measurement-invariance and cross-lagged analyses but were not added to the focal mediation model because their inclusion would estimate a different, fully dynamic panel mediation model and substantially alter the target indirect effects. Thus, the mediation analysis used the longitudinal information required for the hypothesized pathways but did not test every possible wave-specific indirect association (Cole and Maxwell, 2003; Maxwell and Cole, 2007).

Eighth, indirect effects were tested using the bias-corrected bootstrap method with 5,000 resamples. A mediation effect was considered statistically significant if the 95% confidence interval did not include zero. Two specific indirect effects were examined:

Because GAD-7 scores showed slightly greater positive skewness than the other study variables, the focal structural model was additionally evaluated using bias-corrected bootstrap standard errors and 95% confidence intervals based on 5,000 resamples. The direction and statistical significance of all focal paths were compared with the conventional maximum-likelihood estimates to determine whether the slight non-normality materially influenced the SEM inferences.

T1 adherence → T2 emotion regulation difficulties → T3 anxiety symptoms;

T1 adherence → T2 perceived stress → T3 anxiety symptoms.

The total indirect effect of the two parallel mediators was also estimated. In addition, the relative strength of the two indirect effects may be compared to determine whether emotion regulation difficulties or perceived stress represents a stronger explanatory pathway.

Demographic variables, including gender, age, grade, university, academic major/field of study, residence, and socioeconomic status, were included as covariates where appropriate and where complete data were available. Statistical significance was set at *p* < 0.05, and all tests were two-tailed.

## 3 Results

### 3.1 Sample description

The sociodemographic characteristics of the final sample (*N* = 546) are presented in Table 1. The sample included 287 females (52.6%) and 259 males (47.4%). There were 161 freshmen (29.5%), 177 sophomores (32.4%), 128 juniors (23.4%), and 80 seniors (14.7%). Participants had a mean age of 20.09 years (SD = 1.23; range = 18–23 years). University A contributed 286 participants (52.4%) and University B contributed 260 participants (47.6%). The academic-field distribution was 181 students in engineering and technology (33.2%), 117 in health and medical sciences (21.4%), 104 in humanities and social sciences (19.0%), 88 in economics and management (16.1%), and 56 in arts and education (10.3%). Most participants were Han Chinese (*n* = 519, 95.1%), while 27 (4.9%) identified with an ethnic minority group. A total of 294 students (53.8%) reported an urban residence and 252 (46.2%) reported a rural residence. Self-rated family socioeconomic status was low for 112 students (20.5%), middle for 361 (66.1%), and high for 73 (13.4%).

Participant flow and attrition were documented across the three waves. At T1, 680 questionnaires were distributed, 657 were returned (response rate = 96.6%), and 21 were excluded, leaving 636 valid baseline participants. At T2, 594 students participated (retention = 93.4%; dropout = 6.6%), and at T3, 558 participated (retention from baseline = 87.7%; cumulative dropout = 12.3%). After excluding 12 unmatched or invalid longitudinal cases, 546 participants were retained for analysis, corresponding to an analytic retention rate of 85.8%. Participants retained in the final sample did not differ significantly from those not retained on baseline sociodemographic characteristics or ADH, DERS, PSS, and GAD-7 scores (all ps > 0.17; |d| ≤ 0.15; Cramér’s V ≤ 0.07), suggesting that attrition was unlikely to have introduced substantial systematic bias.

Descriptive statistics, normality indices, and internal consistency coefficients for all study variables across the three waves are presented in Table 2. For adherence to traditional Chinese ethnic sports, the mean scores were 3.570 (SD = 0.776) at T1, 3.575 (SD = 0.757) at T2, and 3.580 (SD = 0.765) at T3. For emotion regulation difficulties, the mean scores were 2.911 (SD = 0.765) at T1, 2.909 (SD = 0.772) at T2, and 2.910 (SD = 0.767) at T3. For perceived stress, the mean scores were 1.904 (SD = 0.815) at T1, 1.908 (SD = 0.832) at T2, and 1.885 (SD = 0.813) at T3. For anxiety symptoms, the mean scores were 0.729 (SD = 0.647) at T1, 0.753 (SD = 0.662) at T2, and 0.750 (SD = 0.678) at T3.

**Table 2:** Descriptive statistics, normality indices, and internal consistency (*N* = 546).

Scale	Time	Items	Total M	Total SD	Item-mean M	Item-mean SD	Skewness	Kurtosis	Cronbach α
ADH	T1	23	82.1	17.85	3.57	0.776	−0.354	−0.571	0.959
DERS	T1	36	104.78	27.54	2.911	0.765	0.088	−0.376	0.969
PSS	T1	10	19.04	8.15	1.904	0.815	0.044	−0.603	0.906
GAD	T1	7	5.11	4.53	0.729	0.647	0.852	0.007	0.877
ADH	T2	23	82.23	17.4	3.575	0.757	−0.282	−0.401	0.957
DERS	T2	36	104.73	27.79	2.909	0.772	0.135	−0.663	0.969
PSS	T2	10	19.08	8.32	1.908	0.832	−0.015	−0.537	0.91
GAD	T2	7	5.27	4.63	0.753	0.662	0.818	−0.017	0.889
ADH	T3	23	82.34	17.59	3.58	0.765	−0.216	−0.593	0.958
DERS	T3	36	104.75	27.62	2.91	0.767	0.095	−0.53	0.969
PSS	T3	10	18.85	8.13	1.885	0.813	0.018	−0.585	0.906
GAD	T3	7	5.25	4.75	0.75	0.678	0.981	0.338	0.895

*N* = 546. M, mean; SD, standard deviation; α, Cronbach’s alpha. Total-score and item-mean ranges are provided for interpretability. ADH, adherence to traditional Chinese ethnic sports (23 items; total score 23–115; item-mean score 1–5); DERS, Difficulties in Emotion Regulation Scale (36 items; total score 36–180; item-mean score 1–5); PSS, 10-item Perceived Stress Scale (10 items; total score 0–40; item-mean score 0–4); GAD/GAD-7, 7-item Generalized Anxiety Disorder scale (7 items; total score 0–21; item-mean score 0–3); T1, T2, and T3, Time 1, Time 2, and Time 3.

Skewness ranged from −0.354 to 0.981 and kurtosis ranged from −0.663 to 0.338 across variables and waves. The GAD-7 item-mean scores were slightly more positively skewed (0.818–0.981) than the other variables, but remained below an absolute skewness value of 1.0. Cronbach’s alpha coefficients ranged from 0.877 to 0.969. In the sensitivity analysis using 5,000 bias-corrected bootstrap resamples, all focal paths retained the same direction and statistical-significance pattern as the maximum-likelihood estimates. Differences between the original and bootstrap-based standardized coefficients were no greater than 0.006 in absolute magnitude, and the bootstrap confidence intervals led to identical substantive conclusions. These findings indicated that the slight GAD-7 skewness did not meaningfully bias the SEM standard errors or inferential results.

### 3.2 Correlation analysis of the variables at three time points

**Figure 1:**
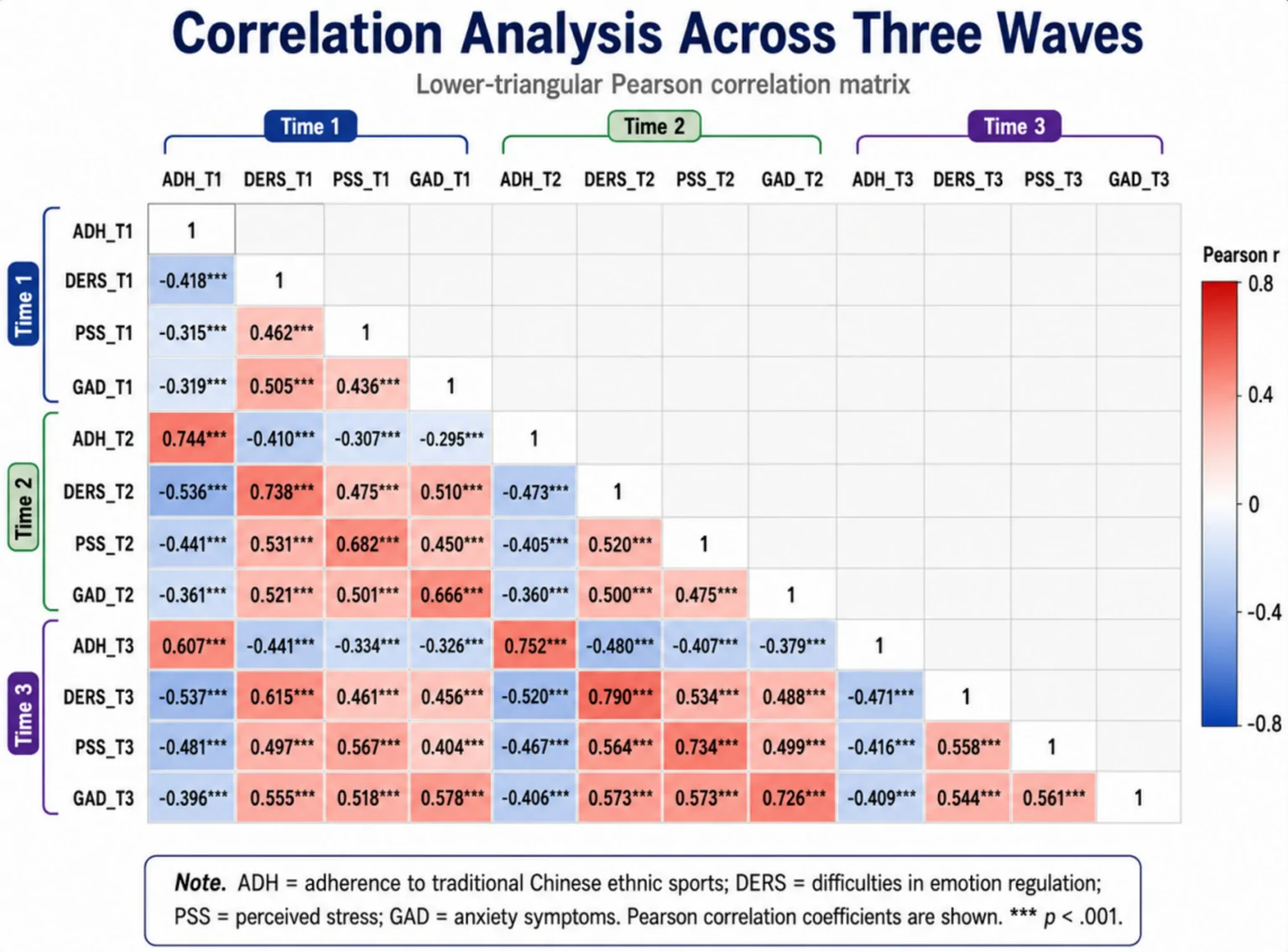
Correlation analysis across three waves (*N* = 546). Values are Pearson correlation coefficients; all displayed off-diagonal coefficients are statistically significant at *p* < 0.001.

Pearson correlations among the study variables across the three waves are visualized in Figure 1; the numerical coefficients are displayed within the lower-triangular correlation matrix. ADH was negatively correlated with DERS, PSS, and GAD-7 anxiety symptoms at all three time points. Specifically, ADH_T1 was negatively associated with DERS_T1, PSS_T1, and GAD_T1, with coefficients ranging from −0.418 to −0.315, all ps < 0.001. Similar negative correlations were observed at T2 and T3, with ADH_T2 correlations ranging from −0.473 to −0.360 and ADH_T3 correlations ranging from −0.471 to −0.409, all ps < 0.001.

The three psychological variables were positively correlated with one another within each wave. At T1, DERS_T1, PSS_T1, and GAD_T1 were positively correlated, with coefficients ranging from 0.436 to 0.505. At T2, DERS_T2, PSS_T2, and GAD_T2 were positively correlated, with coefficients ranging from 0.475 to 0.520. At T3, DERS_T3, PSS_T3, and GAD_T3 were positively correlated, with coefficients ranging from 0.544 to 0.561. All within-wave correlations were statistically significant at *p* < 0.001.

The same constructs showed positive correlations across adjacent and non-adjacent waves. ADH showed significant positive correlations across T1, T2, and T3, with coefficients ranging from 0.607 to 0.752. DERS was positively correlated across waves, with coefficients ranging from 0.615 to 0.790. PSS showed positive cross-wave correlations ranging from 0.567 to 0.734, and GAD showed positive cross-wave correlations ranging from 0.578 to 0.726. All cross-wave correlations for the same constructs were statistically significant at *p* < 0.001.

Cross-variable longitudinal correlations were also observed. Earlier ADH scores were negatively correlated with later DERS, PSS, and GAD scores. For example, ADH_T1 was negatively correlated with DERS_T2, PSS_T2, and GAD_T2, with coefficients of −0.536, −0.441, and −0.361, respectively, and with DERS_T3, PSS_T3, and GAD_T3, with coefficients of −0.537, −0.481, and −0.396, respectively. In contrast, DERS, PSS, and GAD were generally positively correlated with one another across waves, with correlation coefficients ranging from 0.404 to 0.573, all ps < 0.001.

### 3.3 Common method Bias test

Harman’s single-factor test was conducted to assess common method bias, and the results are presented in Table 3. For the Time 1 items, the KMO value was 0.976, and Bartlett’s test of sphericity was significant, χ^2^(2850) = 24238.740, *p* < 0.001. The first unrotated factor had an eigenvalue of 24.328 and explained 32.010% of the total variance. For the Time 2 items, the KMO value was 0.978, with a significant Bartlett’s test, χ^2^(2850) = 24302.732, *p* < 0.001. The first factor had an eigenvalue of 25.510 and explained 33.566% of the total variance. For the Time 3 items, the KMO value was 0.978, and Bartlett’s test was also significant, χ^2^(2850) = 24429.747, *p* < 0.001. The first factor had an eigenvalue of 26.002 and accounted for 34.214% of the total variance.

**Table 3:** Harman’s single-factor test for common method bias (*N* = 546).

Scope	KMO	Bartlett χ^2^	df	*p*	First eigenvalue	Variance explained by first factor (%)	No. of factors with eigenvalue > 1	Interpretation
Time 1 items	0.976	24238.740	2,850	<0.001	24.328	32.010	4	No dominant single-factor pattern
Time 2 items	0.978	24302.732	2,850	<0.001	25.510	33.566	4	No dominant single-factor pattern
Time 3 items	0.978	24429.747	2,850	<0.001	26.002	34.214	4	No dominant single-factor pattern
All item variables across T1–T3	0.966	87082.271	25,878	<0.001	68.702	30.132	32	No dominant single-factor pattern

*N* = 546. KMO, Kaiser–Meyer–Olkin measure; CMB, common method bias. A first-factor variance below 40% was interpreted as indicating no serious single-factor common method bias. ADH, adherence to traditional Chinese ethnic sports (23 items; total score 23–115; item-mean score 1–5); DERS, Difficulties in Emotion Regulation Scale (36 items; total score 36–180; item-mean score 1–5); PSS, 10-item Perceived Stress Scale (10 items; total score 0–40; item-mean score 0–4); GAD/GAD-7, 7-item Generalized Anxiety Disorder scale (7 items; total score 0–21; item-mean score 0–3); T1, T2, and T3 = Time 1, Time 2, and Time 3. The first-factor percentage is a descriptive diagnostic only; it does not rule out residual common method variance.

When all item-level variables across the three waves were entered simultaneously, the KMO value was 0.966, and Bartlett’s test of sphericity was significant, χ^2^(25878) = 87082.271, *p* < 0.001. The first unrotated factor had an eigenvalue of 68.702 and explained 30.132% of the total variance. The number of factors with eigenvalues greater than 1 was 4 at each individual time point and 32 when all item-level variables across T1–T3 were included. Across all analyses, the variance explained by the first factor ranged from 30.132% to 34.214%.

Full collinearity VIF diagnostics were conducted to further examine common method bias, and the results are presented in Table 4. At Time 1, the R^2^ values from the full-collinearity regressions ranged from 0.202 to 0.376, and the corresponding VIF values ranged from 1.254 to 1.602. At Time 2, the R^2^ values ranged from 0.267 to 0.407, with VIF values ranging from 1.364 to 1.685. At Time 3, the R^2^ values ranged from 0.271 to 0.431, and the corresponding VIF values ranged from 1.371 to 1.757.

**Table 4:** Full-collinearity VIF test for common method bias (*N* = 546).

Wave	Variable	*R* ^2^	VIF
Time 1	ADH	0.202	1.254
Time 1	DERS	0.376	1.602
Time 1	PSS	0.279	1.388
Time 1	GAD	0.315	1.459
Time 2	ADH	0.267	1.364
Time 2	DERS	0.407	1.685
Time 2	PSS	0.352	1.544
Time 2	GAD	0.322	1.474
Time 3	ADH	0.271	1.371
Time 3	DERS	0.431	1.757
Time 3	PSS	0.418	1.719
Time 3	GAD	0.404	1.678

*N* = 546. *R*^2^, coefficient of determination; VIF, variance inflation factor; CMB, common method bias. The criterion and interpretation columns were removed because the same rule applies to every row: VIF < 3.30 indicates no serious full-collinearity common method bias. This diagnostic should not be interpreted as ruling out all forms of common method variance.

Across all three waves, VIF values for ADH, DERS, PSS, and GAD-7 ranged from 1.254 to 1.757, below the 3.30 diagnostic criterion. Considered together, the Harman and full-collinearity results did not indicate a dominant single factor or problematic full collinearity. Nevertheless, because both procedures have limited sensitivity and all focal constructs were measured by self-report, the analyses cannot exclude residual common method variance.

### 3.4 Longitudinal measurement invariance

Longitudinal measurement invariance across the three waves was examined for ADH, DERS, PSS, and GAD, and the results are presented in Table 5. For ADH, the configural model showed excellent fit, χ^2^(24) = 19.530, CFI = 1.000, TLI = 1.001, RMSEA = 0.000, and SRMR = 0.007. After imposing equality constraints on factor loadings, intercepts, and residual variances, the metric, scalar, and strict models continued to show excellent fit, with CFI values of 1.000 and RMSEA values of 0.000. The changes in CFI, RMSEA, and SRMR across nested models were minimal.

**Table 5:** Longitudinal measurement invariance across three waves (N = 546).

Scale	Model	χ^2^	df	CFI	TLI	RMSEA	SRMR	ΔCFI	ΔRMSEA	ΔSRMR
ADH	Configural	19.530	24	1.000	1.001	0.000	0.007	—	—	—
ADH	Metric	21.672	28	1.000	1.001	0.000	0.007	0.000	0.000	0.001
ADH	Scalar	31.010	32	1.000	1.000	0.000	0.007	0.000	0.000	0.000
ADH	Strict	36.095	38	1.000	1.000	0.000	0.007	0.000	0.000	−0.001
DERS	Configural	31.365	24	0.999	0.998	0.024	0.008	—	—	—
DERS	Metric	34.680	28	0.999	0.999	0.021	0.008	0.000	−0.003	0.000
DERS	Scalar	34.979	32	1.000	1.000	0.013	0.008	0.001	−0.008	0.000
DERS	Strict	40.119	38	1.000	1.000	0.010	0.008	0.000	−0.003	0.000
PSS	Configural	22.823	24	1.000	1.000	0.000	0.014	—	—	—
PSS	Metric	23.783	28	1.000	1.001	0.000	0.014	0.000	0.000	0.000
PSS	Scalar	27.272	32	1.000	1.001	0.000	0.014	0.000	0.000	0.000
PSS	Strict	31.259	38	1.000	1.002	0.000	0.014	0.000	0.000	0.000
GAD	Configural	24.029	24	1.000	1.000	0.001	0.015	—	—	—
GAD	Metric	36.544	28	0.998	0.997	0.024	0.016	−0.002	0.022	0.001
GAD	Scalar	41.146	32	0.997	0.997	0.023	0.016	0.000	−0.001	0.000
GAD	Strict	56.035	38	0.995	0.995	0.030	0.020	−0.002	0.007	0.005

*N* = 546. CFI, comparative fit index; TLI, Tucker–Lewis index; RMSEA, root mean square error of approximation; SRMR, standardized root mean square residual; Δ = change between nested models. Longitudinal invariance was considered supported when ΔCFI ≤ 0.010 and ΔRMSEA ≤ 0.015.

For DERS, the configural model also showed good fit, χ^2^(24) = 31.365, CFI = 0.999, TLI = 0.998, RMSEA = 0.024, and SRMR = 0.008. The metric, scalar, and strict models showed CFI values ranging from 0.999 to 1.000, TLI values ranging from 0.999 to 1.000, RMSEA values ranging from 0.010 to 0.021, and SRMR values of 0.008. The changes in fit indices across increasingly constrained models were small, with ΔCFI ranging from 0.000 to 0.001 and ΔRMSEA ranging from −0.008 to −0.003.

For PSS, the configural model demonstrated good fit, χ^2^(24) = 22.823, CFI = 1.000, TLI = 1.000, RMSEA = 0.000, and SRMR = 0.014. The metric, scalar, and strict models also showed good fit, with CFI values of 1.000, TLI values ranging from 1.001 to 1.002, RMSEA values of 0.000, and SRMR values of 0.014. No changes were observed in CFI, RMSEA, or SRMR across the constrained models.

For GAD, the configural model showed good fit, χ^2^(24) = 24.029, CFI = 1.000, TLI = 1.000, RMSEA = 0.001, and SRMR = 0.015. The metric, scalar, and strict models also demonstrated acceptable fit, with CFI values ranging from 0.995 to 0.998, TLI values ranging from 0.995 to 0.997, RMSEA values ranging from 0.023 to 0.030, and SRMR values ranging from 0.016 to 0.020. Across the nested GAD models, ΔCFI values ranged from −0.002 to 0.000, ΔRMSEA values ranged from −0.001 to 0.022, and ΔSRMR values ranged from 0.000 to 0.005.

Overall, the fit indices for the configural, metric, scalar, and strict invariance models were reported across all four constructs. The ΔCFI values were within the specified criterion of 0.010 for all constrained models, while most ΔRMSEA values were within the specified criterion of 0.015, with the exception of the GAD metric model, which showed a ΔRMSEA of 0.022.

### 3.5 Cross-lagged panel analysis

The results of the cross-lagged panel model are presented in Table 6. All autoregressive paths were statistically significant across adjacent waves. Specifically, ADH showed significant stability from T1 to T2, *β* = 0.687, SE = 0.032, t = 21.710, *p* < 0.001, and from T2 to T3, β = 0.655, SE = 0.032, t = 20.429, *p* < 0.001. DERS also showed significant autoregressive effects from T1 to T2, β = 0.528, SE = 0.033, t = 15.958, *p* < 0.001, and from T2 to T3, *β* = 0.624, SE = 0.032, t = 19.409, *p* < 0.001. Similar autoregressive effects were observed for PSS from T1 to T2, β = 0.507, SE = 0.034, t = 15.052, *p* < 0.001, and from T2 to T3, *β* = 0.543, SE = 0.033, t = 16.203, *p* < 0.001. GAD also showed significant stability from T1 to T2, β = 0.476, SE = 0.036, t = 13.305, *p* < 0.001, and from T2 to T3, *β* = 0.517, SE = 0.032, *t* = 16.090, *p* < 0.001.

**Table 6:** Cross-lagged panel model analysis (N = 546).

Type	Path	*β*	*SE*	*t*	*p*
Autoregressive	ADH_T1 → ADH_T2	0.687	0.032	21.71	<0.001
Autoregressive	DERS_T1 → DERS_T2	0.528	0.033	15.958	<0.001
Autoregressive	PSS_T1 → PSS_T2	0.507	0.034	15.052	<0.001
Autoregressive	GAD_T1 → GAD_T2	0.476	0.036	13.305	<0.001
Autoregressive	ADH_T2 → ADH_T3	0.655	0.032	20.429	<0.001
Autoregressive	DERS_T2 → DERS_T3	0.624	0.032	19.409	<0.001
Autoregressive	PSS_T2 → PSS_T3	0.543	0.033	16.203	<0.001
Autoregressive	GAD_T2 → GAD_T3	0.517	0.032	16.09	<0.001
Cross-lagged	DERS_T1 → ADH_T2	−0.099	0.036	−2.774	0.006
Cross-lagged	PSS_T1 → ADH_T2	−0.041	0.033	−1.237	0.217
Cross-lagged	GAD_T1 → ADH_T2	−0.008	0.034	−0.223	0.823
Cross-lagged	ADH_T1 → DERS_T2	−0.244	0.029	−8.337	<0.001
Cross-lagged	PSS_T1 → DERS_T2	0.101	0.031	3.271	0.001
Cross-lagged	GAD_T1 → DERS_T2	0.121	0.032	3.831	<0.001
Cross-lagged	ADH_T1 → PSS_T2	−0.18	0.032	−5.608	<0.001
Cross-lagged	DERS_T1 → PSS_T2	0.181	0.036	5.01	<0.001
Cross-lagged	GAD_T1 → PSS_T2	0.079	0.035	2.294	0.022
Cross-lagged	ADH_T1 → GAD_T2	−0.082	0.033	−2.475	0.014
Cross-lagged	DERS_T1 → GAD_T2	0.156	0.037	4.175	<0.001
Cross-lagged	PSS_T1 → GAD_T2	0.196	0.035	5.617	<0.001
Cross-lagged	DERS_T2 → ADH_T3	−0.111	0.036	−3.126	0.002
Cross-lagged	PSS_T2 → ADH_T3	−0.055	0.034	−1.6	0.110
Cross-lagged	GAD_T2 → ADH_T3	−0.061	0.033	−1.825	0.069
Cross-lagged	ADH_T2 → DERS_T3	−0.155	0.029	−5.339	<0.001
Cross-lagged	PSS_T2 → DERS_T3	0.117	0.031	3.793	<0.001
Cross-lagged	GAD_T2 → DERS_T3	0.064	0.03	2.142	0.033
Cross-lagged	ADH_T2 → PSS_T3	−0.129	0.031	−4.084	<0.001
Cross-lagged	DERS_T2 → PSS_T3	0.165	0.035	4.711	<0.001
Cross-lagged	GAD_T2 → PSS_T3	0.112	0.033	3.432	<0.001
Cross-lagged	ADH_T2 → GAD_T3	−0.048	0.031	−1.543	0.123
Cross-lagged	DERS_T2 → GAD_T3	0.181	0.034	5.267	<0.001
Cross-lagged	PSS_T2 → GAD_T3	0.214	0.033	6.522	<0.001

*N* = 546. *β*, standardized coefficient; SE, standard error. Autoregressive paths represent construct stability across adjacent waves, whereas cross-lagged paths represent prospective associations between different constructs after controlling for prior levels. Statistical significance was set at *p* < 0.05.

For the cross-lagged paths from T1 to T2, ADH_T1 was significantly and inversely associated with DERS_T2, *β* = −0.244, SE = 0.029, *t* = −8.337, *p* < 0.001; PSS_T2, β = −0.180, SE = 0.032, *t* = −5.608, *p* < 0.001; and GAD_T2, β = −0.082, SE = 0.033, *t* = −2.475, *p* = 0.014. DERS_T1 was significantly and inversely associated with ADH_T2, *β* = −0.099, SE = 0.036, *t* = −2.774, *p* = 0.006, and positively associated with PSS_T2, *β* = 0.181, SE = 0.036, *t* = 5.010, *p* < 0.001, and GAD_T2, β = 0.156, SE = 0.037, *t* = 4.175, *p* < 0.001. PSS_T1 was positively associated with DERS_T2, *β* = 0.101, SE = 0.031, *t* = 3.271, *p* = 0.001, and GAD_T2, β = 0.196, SE = 0.035, *t* = 5.617, *p* < 0.001, but was not significantly associated with ADH_T2, *β* = −0.041, SE = 0.033, *t* = −1.237, *p* = 0.217. GAD_T1 was positively associated with DERS_T2, *β* = 0.121, SE = 0.032, *t* = 3.831, *p* < 0.001, and PSS_T2, *β* = 0.079, SE = 0.035, *t* = 2.294, *p* = 0.022, but was not significantly associated with ADH_T2, *β* = −0.008, SE = 0.034, *t* = −0.223, *p* = 0.823.

**Figure 2:**
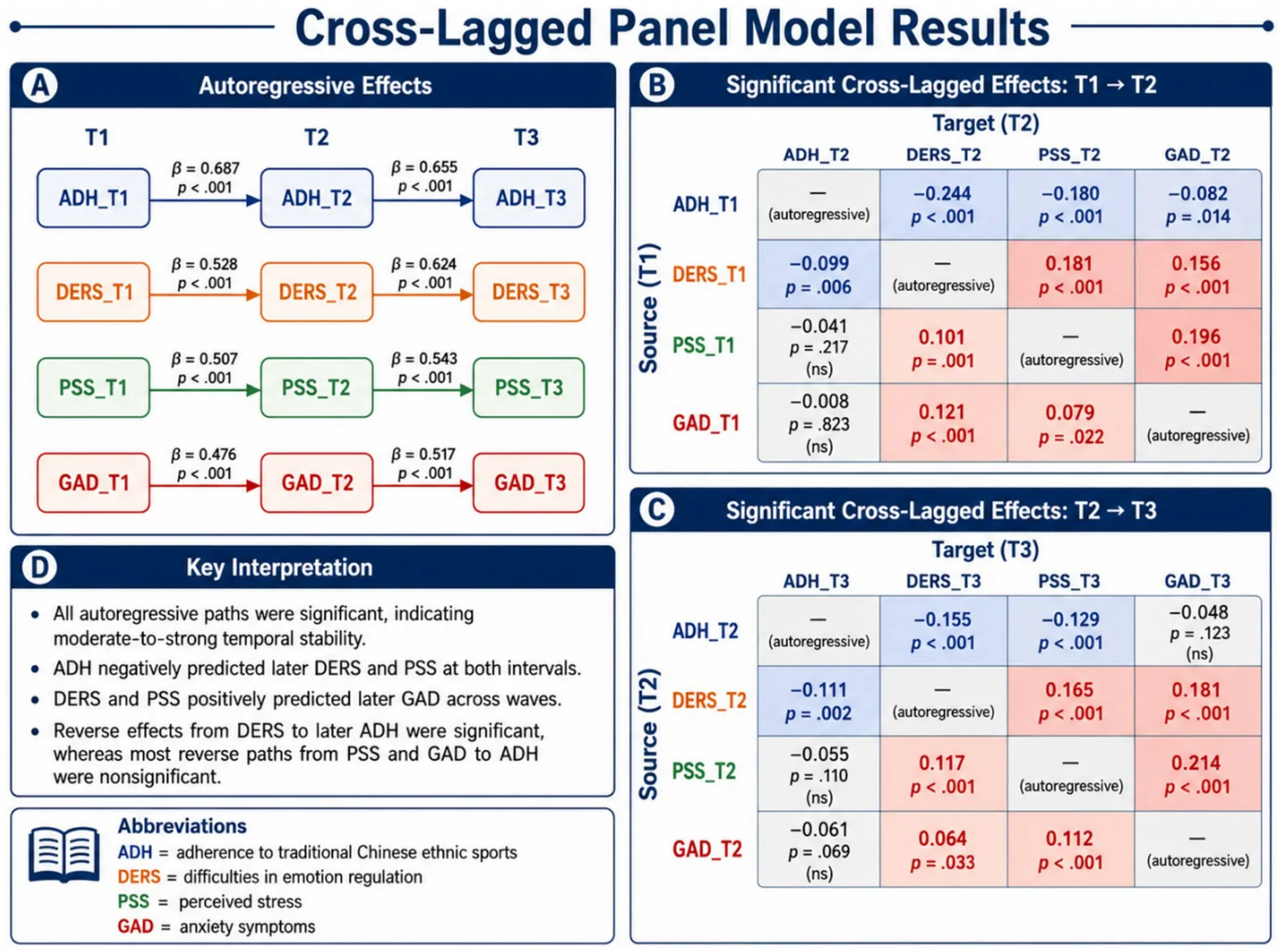
Cross-lagged panel model and data interpretation diagram.

For the cross-lagged paths from T2 to T3, ADH_T2 was significantly and inversely associated with DERS_T3, *β* = −0.155, SE = 0.029, *t* = −5.339, *p* < 0.001, and PSS_T3, *β* = −0.129, SE = 0.031, *t* = −4.084, *p* < 0.001. The association between ADH_T2 and GAD_T3 was not statistically significant, *β* = −0.048, SE = 0.031, *t* = −1.543, *p* = 0.123. DERS_T2 was significantly and inversely associated with ADH_T3, *β* = −0.111, SE = 0.036, *t* = −3.126, *p* = 0.002, and positively associated with PSS_T3, *β* = 0.165, SE = 0.035, *t* = 4.711, *p* < 0.001, and GAD_T3, *β* = 0.181, SE = 0.034, *t* = 5.267, *p* < 0.001. PSS_T2 was positively associated with DERS_T3, *β* = 0.117, SE = 0.031, *t* = 3.793, *p* < 0.001, and GAD_T3, β = 0.214, SE = 0.033, *t* = 6.522, *p* < 0.001, but was not significantly associated with ADH_T3, *β* = −0.055, SE = 0.034, *t* = −1.600, *p* = 0.110. GAD_T2 was positively associated with DERS_T3, *β* = 0.064, SE = 0.030, *t* = 2.142, *p* = 0.033, and PSS_T3, *β* = 0.112, SE = 0.033, *t* = 3.432, *p* < 0.001. The association between GAD_T2 and ADH_T3 was not statistically significant, *β* = −0.061, SE = 0.033, *t* = −1.825, *p* = 0.069 (see Figure 2).

### 3.6 Longitudinal parallel mediation analysis

**Figure 3:**
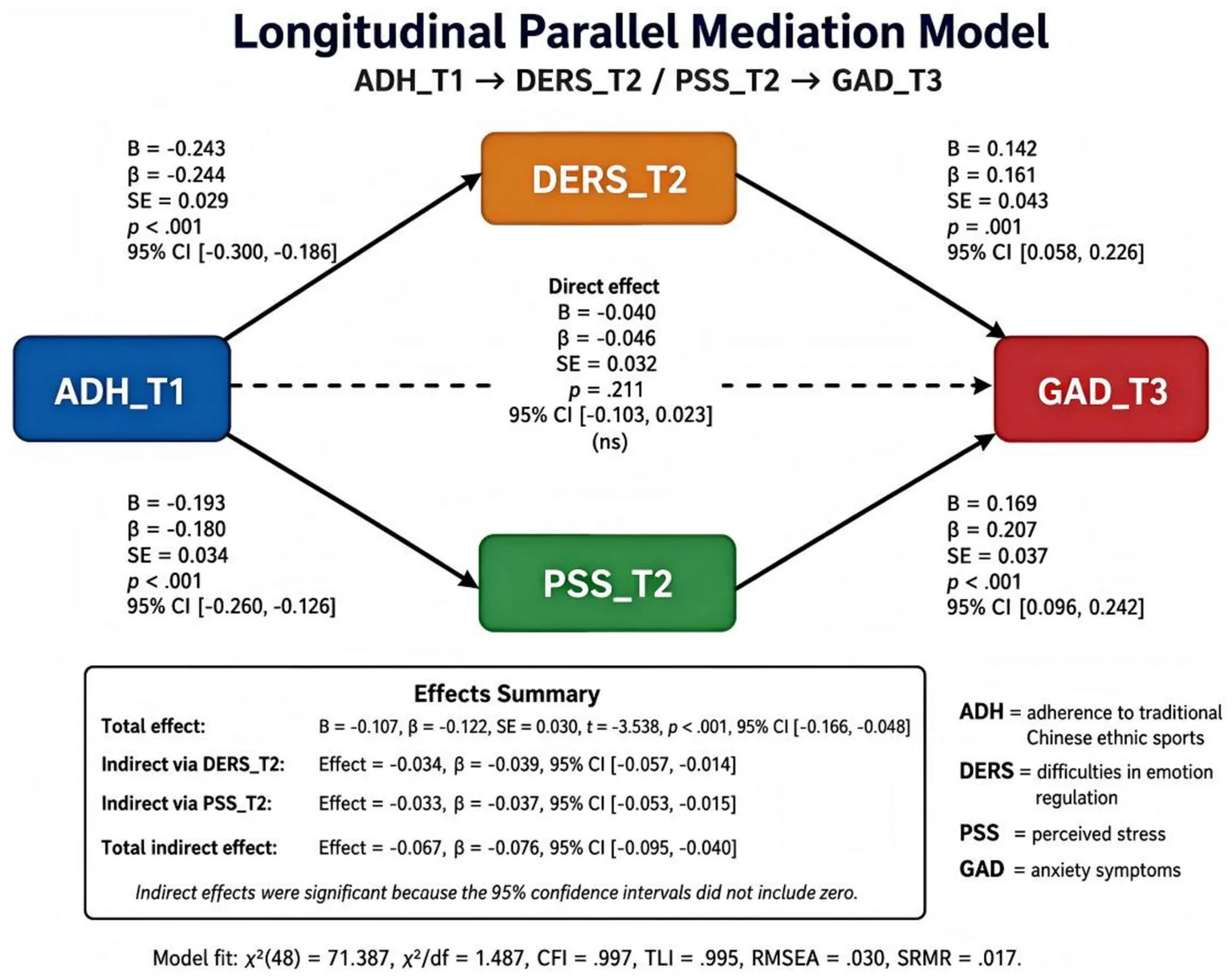
Longitudinal parallel mediation model (*N* = 546). Solid arrows represent statistically significant effects, whereas the dashed arrow represents the nonsignificant direct effect from ADH_T1 to GAD_T3 after inclusion of the two parallel mediators. Standardized path coefficients are displayed.

The results of the longitudinal parallel mediation model are presented in Table 7 and Figure 3. The longitudinal parallel mediation model demonstrated a good overall fit to the data: χ^2^(48) = 71.387, *p* = 0.016, χ^2^/df = 1.487, CFI = 0.997, TLI = 0.995, RMSEA = 0.030, 90% CI [0.013, 0.044], and SRMR = 0.017. Although the chi-square test was statistically significant, this result is common in relatively large samples. The remaining fit indices all met commonly recommended criteria, indicating that the proposed longitudinal parallel mediation model fitted the data well. In the path coefficient model, ADH_T1 was significantly and inversely associated with DERS_T2 at the subsequent wave, *B* = −0.243, SE = 0.029, *t* = −8.337, *p* < 0.001, *β* = −0.244, 95% CI [−0.300, −0.186]. ADH_T1 was also significantly and inversely associated with PSS_T2 at the subsequent wave, *B* = −0.193, SE = 0.034, *t* = −5.608, *p* < 0.001, *β* = −0.180, 95% CI [−0.260, −0.126]. In turn, DERS_T2 was significantly and positively associated with GAD_T3 at the subsequent wave, *B* = 0.142, SE = 0.043, *t* = 3.261, *p* = 0.001, *β* = 0.161, 95% CI [0.058, 0.226]. PSS_T2 was also significantly and positively associated with GAD_T3 at the subsequent wave, *B* = 0.169, SE = 0.037, *t* = 4.582, *p* < 0.001, *β* = 0.207, 95% CI [0.096, 0.242].

**Table 7:** Longitudinal parallel mediation model and bootstrap indirect effects (*N* = 546).

Effect type	Path	B / Effect	SE	t	*p*	*β*	95% CI
Path coefficient	ADH_T1 → DERS_T2	−0.243	0.029	−8.337	<0.001	−0.244	[−0.300, −0.186]
Path coefficient	ADH_T1 → PSS_T2	−0.193	0.034	−5.608	<0.001	−0.180	[−0.260, −0.126]
Path coefficient	DERS_T2 → GAD_T3	0.142	0.043	3.261	0.001	0.161	[0.058, 0.226]
Path coefficient	PSS_T2 → GAD_T3	0.169	0.037	4.582	<0.001	0.207	[0.096, 0.242]
Direct effect	ADH_T1 → GAD_T3	−0.040	0.032	−1.252	0.211	−0.046	[−0.103, 0.023]
Total effect	ADH_T1 → GAD_T3	−0.107	0.030	−3.538	<0.001	−0.122	[−0.166, −0.048]
Indirect effect	ADH_T1 → DERS_T2 → GAD_T3	−0.034	—	—	Significant	−0.039	[−0.057, −0.014]
Indirect effect	ADH_T1 → PSS_T2 → GAD_T3	−0.033	—	—	Significant	−0.037	[−0.053, −0.015]
Total indirect effect	ADH_T1 → DERS_T2 / PSS_T2 → GAD_T3	−0.067	—	—	Significant	−0.076	[−0.095, −0.040]

*N* = 546. B, unstandardized coefficient/effect; β, standardized coefficient/effect; SE, standard error; CI, confidence interval. Indirect-effect CIs were estimated using 5,000 bias-corrected bootstrap resamples and were considered significant when the 95% CI excluded zero. Baseline DERS, PSS, and GAD-7 scores were controlled. DERS_T2 and PSS_T2 were specified as parallel mediators; In this observational study, the terms direct, indirect, and total effect refer to model-based path decompositions and do not imply experimentally established causality.

The direct effect of ADH_T1 on GAD_T3 was not statistically significant after including DERS_T2 and PSS_T2 as parallel mediators, *B* = −0.040, SE = 0.032, *t* = −1.252, *p* = 0.211, *β* = −0.046, 95% CI [−0.103, 0.023]. The total effect of ADH_T1 on GAD_T3 was statistically significant, B = −0.107, SE = 0.030, *t* = −3.538, *p* < 0.001, *β* = −0.122, 95% CI [−0.166, −0.048].

Bootstrap analyses with 5,000 resamples were conducted to test the indirect effects. The indirect effect through DERS_T2 was significant, Effect = −0.034, *β* = −0.039, 95% CI [−0.057, −0.014]. The indirect effect through PSS_T2 was also significant, Effect = −0.033, *β* = −0.037, 95% CI [−0.053, −0.015]. The total indirect effect through the two parallel mediators was significant, Effect = −0.067, *β* = −0.076, 95% CI [−0.095, −0.040]. The standardized indirect associations were similar and modest in magnitude.

## 4 Discussion

### 4.1 Principal findings

This study examined longitudinal associations among overall adherence to traditional Chinese ethnic sports, emotion regulation difficulties, perceived stress, and anxiety symptoms among college students. The autoregressive coefficients were moderate to strong across adjacent waves (*β* = 0.476–0.687 from T1 to T2 and *β* = 0.517–0.655 from T2 to T3), indicating substantial continuity in all four constructs (Cole and Maxwell, 2003; Hamaker et al., 2015). The prospective associations from adherence to later emotion regulation difficulties (*β* = −0.244 and −0.155) and perceived stress (*β* = −0.180 and −0.129) were small to moderate, while emotion regulation difficulties and perceived stress were positively associated with later anxiety symptoms (Gratz and Roemer, 2004; Cohen et al., 1983; Sloan et al., 2017). In the parallel mediation model, the two standardized indirect associations were similar and modest (*β* = −0.039 through DERS and *β* = −0.037 through PSS; total indirect *β* = −0.076), whereas the direct path was nonsignificant (*β* = −0.046). The findings therefore indicate statistically reliable but not large prospective associations and should be interpreted as explanatory evidence at the panel level rather than as proof of causal mechanisms (Cole and Maxwell, 2003; Maxwell and Cole, 2007).

The principal theoretical value of these findings lies less in proposing unprecedented bivariate relationships than in specifying the role of adherence quality within the physical activity–mental health literature. Previous intervention studies and reviews have reported that Tai Chi, Qigong, and related traditional Chinese exercises are associated with improvements in anxiety and depressive symptoms among college students (Du et al., 2022; Lin et al., 2022; Qi et al., 2022), but participation-based measures provide limited information about whether the intended exercise prescription was understood, enacted, and sustained. By operationalizing adherence as a multidimensional implementation construct, the present study identifies a candidate boundary condition that may help explain heterogeneity in psychological outcomes among students who have similar opportunities to participate (Rhodes and Fiala, 2009; Garber et al., 2011; He et al., 2025). This represents an incremental but conceptually useful extension of prior adherence research rather than a claim that the individual adherence–emotion–stress–anxiety links are entirely new.

### 4.2 Temporal stability and cross-lagged associations

The substantial autoregressive stability has two implications. First, controlling prior scores was necessary because students maintained relatively stable rank ordering in adherence and psychological functioning across the academic year (Cole and Maxwell, 2003; Maxwell and Cole, 2007). Second, this stability increases the possibility that conventional CLPM coefficients partly reflect enduring between-person differences rather than purely within-person fluctuations (Hamaker et al., 2015). The cross-lagged estimates therefore show whether students who were relatively higher or lower on one construct tended to differ on another construct at the subsequent wave, conditional on prior scores. They offer stronger temporal information than cross-sectional correlations, but they do not establish that a within-person increase in adherence would necessarily be followed by a within-person reduction in stress or anxiety. Thus, the present cross-lagged paths characterize prospective differences in relative standing across students rather than person-specific change processes.

Adherence was inversely associated with later emotion regulation difficulties and perceived stress across both adjacent intervals, whereas the direct association with anxiety was inconsistent: the ADH_T1 → GAD_T2 path was small but significant (*β* = −0.082), while the ADH_T2 → GAD_T3 path was nonsignificant (*β* = −0.048). Rather than treating these findings as contradictory, the pattern suggests that the direct association may be weaker and more sensitive to the timing of assessment or semester-specific demands than the associations with the proposed mediators. It is also consistent with the possibility that adherence is related to anxiety primarily through intervening regulatory and appraisal processes, although the mediation model cannot establish that those processes are causal (Cole and Maxwell, 2003; Maxwell and Cole, 2007).

The reverse associations were selective rather than uniform. Greater emotion regulation difficulties were prospectively associated with lower later adherence at both intervals (*β* = −0.099 and −0.111), whereas perceived stress and anxiety symptoms were not consistently associated with subsequent adherence. One interpretation is that difficulties maintaining goal-directed behavior and controlling impulses under distress may also interfere with planning, persistence, and adjustment of an exercise prescription (Gratz and Roemer, 2004; Sloan et al., 2017). However, the magnitudes were small and the conventional CLPM does not separate within-person dynamics from stable between-person differences. RI-CLPM or related multilevel panel models are needed to determine whether occasions on which a student experiences more emotion dysregulation than usual are followed by lower-than-usual adherence (Hamaker et al., 2015; Mulder and Hamaker, 2021).

### 4.3 Adherence and emotion regulation difficulties

Overall adherence was prospectively associated with fewer later emotion regulation difficulties, with the association decreasing from *β* = −0.244 in the first interval to *β* = −0.155 in the second. This pattern is consistent with emotion regulation theory, which defines adaptive regulation in terms of awareness, understanding, acceptance, modulation, impulse control, and goal-directed behavior under distress (Gross, 1998; Gratz and Roemer, 2004). At the same time, the association should not be interpreted as evidence that exercise adherence directly changes emotion-regulation capacity. The self-regulatory component of the adherence scale may itself capture persistence and adaptive adjustment, creating theoretically meaningful but potentially overlapping self-regulatory content with DERS. Future analyses of the cognitive, behavioral, and self-regulatory adherence dimensions separately would help determine which component is most strongly associated with later emotion regulation difficulties.

Traditional Chinese ethnic sports offer several plausible contexts for this association because they combine movement control, breathing, attentional focus, rhythm, bodily awareness, and culturally meaningful practice (Wayne and Kaptchuk, 2008; Qi et al., 2022). Students with higher adherence may encounter these regulatory demands more frequently and consistently, providing repeated opportunities to notice internal states and maintain goal-directed control during discomfort (Gross, 1998; Sheng et al., 2024). Nevertheless, the present self-report design cannot identify which active component—breathing, attentional training, movement mastery, social context, or routine—accounts for the association. Component-specific interventions and objective practice records are needed before these processes can be described as causal mechanisms.

The distinction between participation and adherence is therefore more than a terminological refinement. Participation indicates exposure, whereas the Ethnic Traditional Sports Exercise Prescription Adherence Scale assesses whether students cognitively understand, behaviorally execute, and self-regulate an exercise prescription grounded in FITT-VP principles (He et al., 2025). Exercise-prescription research similarly emphasizes that benefits depend on structured and sustained implementation rather than unsystematic exposure alone (Garber et al., 2011; Rhodes and Fiala, 2009). This distinction may help explain why course enrollment does not produce uniform psychological outcomes. However, use of an overall composite also means that the current study cannot determine whether cognitive understanding, behavioral execution, or self-regulatory adherence is the most consequential dimension.

### 4.4 Adherence and perceived stress

The perceived-stress pathway was also statistically reliable but modest. Adherence was prospectively associated with lower perceived stress (*β* = −0.180 and −0.129), and perceived stress showed the larger of the two mediator-to-anxiety coefficients in the focal model (*β* = 0.207). Stress appraisal theory suggests that this pattern may reflect differences in how students evaluate the controllability and manageability of academic and interpersonal demands rather than changes in the objective number of stressors they encounter (Folkman et al., 1986; Lazarus, 1993). The PSS captures perceived unpredictability, uncontrollability, and overload (Cohen et al., 1983); thus, the findings concern subjective appraisal and should not be interpreted as evidence that adherence removes external stressors.

This interpretation is consistent with prior evidence linking physical activity to students’ stress and recovery. A daily-level longitudinal study found that leisure-time physical activity and activity breaks were associated with aspects of stress load and recovery among university students (Teuber et al., 2024), while broader reviews have emphasized reciprocal and context-dependent links among physical activity, stress, and academic functioning (Wunsch et al., 2021). The present study extends this literature by focusing on multidimensional adherence over longer academic intervals rather than on the occurrence or duration of activity alone. Repeated practice, bodily mastery, routine, and temporary psychological detachment may be relevant correlates of coping appraisal (VanKim and Nelson, 2013; Folkman et al., 1986; Lazarus, 1993; Zhao et al., 2023), but the current measures do not permit these possibilities to be separated.

Perceived stress was positively associated with later anxiety symptoms, consistent with the proposition that appraising demands as uncontrollable or overwhelming is related to worry, arousal, and avoidance (Cohen et al., 1983; Folkman et al., 1986; Lazarus, 1993; Zhao et al., 2023). Nevertheless, the standardized indirect association through PSS was only *β* = −0.037. The result is therefore theoretically informative but should not be equated with a large or clinically sufficient reduction in anxiety. Adherence is likely to be one of several behavioral and contextual factors associated with students’ stress and anxiety, alongside academic demands, social resources, financial strain, sleep, and access to mental health care (VanKim and Nelson, 2013; Wunsch et al., 2021).

### 4.5 Parallel mediating roles of emotion regulation difficulties and perceived stress

Emotion regulation difficulties and perceived stress showed two significant parallel indirect associations of nearly identical magnitude (*β* = −0.039 and −0.037). Their combined standardized indirect association was modest (*β* = −0.076), and the direct ADH_T1 → GAD_T3 path was nonsignificant after both mediators were included. This pattern should not be labeled “full mediation,” nor does it demonstrate that adherence causes changes in the mediators or anxiety. In an observational model, a nonsignificant direct path can coexist with unmeasured confounding, measurement error, or alternative pathways. The findings more cautiously support two temporally separated explanatory routes that are consistent with emotion regulation and stress appraisal theories (Cole and Maxwell, 2003; Maxwell and Cole, 2007).

The two mediators represent related but distinguishable processes. Emotion regulation difficulties reflect problems in identifying, accepting, and managing emotional responses, whereas perceived stress reflects the appraisal of life demands as uncontrollable or excessive (Cohen et al., 1983; Gratz and Roemer, 2004). The findings indicate that higher adherence was prospectively associated with lower later anxiety symptoms in two model-based indirect associations involving lower subsequent emotion regulation difficulties and lower subsequent perceived stress (Gross, 1998; Lazarus, 1993). Because DERS and PSS were measured concurrently at T2, the analysis deliberately treated them as parallel rather than sequential mediators; therefore, the results support two independent indirect pathways and do not imply that one mediator temporally precedes the other. The near-equivalent standardized indirect associations also indicate that neither pathway clearly dominated the other in the present sample, while their concurrent T2 assessment prevents any claim about temporal ordering between DERS and PSS. Accordingly, these indirect effects should be understood as temporally ordered statistical associations compatible with the proposed theories, not as evidence that adherence produces psychological improvement or anxiety reduction.

Taken together, the parallel findings provide a more differentiated account of the association between traditional Chinese ethnic sports and mental health. When traditional sports are practiced with sustained cognitive, behavioral, and self-regulatory adherence, students may receive more consistent exposure to bodily discipline, attentional control, breathing, rhythm, and culturally embedded engagement (Wayne and Kaptchuk, 2008; Qi et al., 2022; Garber et al., 2011; He et al., 2025). The study therefore identifies adherence quality as a plausible organizing construct linking program implementation with two psychological domains. It does not, however, identify which specific practice ingredient is active or establish that the indirect pathways are causal.

### 4.6 Theoretical and practical implications

The theoretical contribution can be summarized in three points. First, the study reframes adherence as implementation quality rather than as a synonym for participation, thereby introducing a potential boundary condition into models of physical activity and mental health (Rhodes and Fiala, 2009; Garber et al., 2011; He et al., 2025). Second, it integrates two conceptually distinct domains emotion regulatory capacity and cognitive stress appraisal as parallel, similarly sized explanatory pathways (Gross, 1998; Folkman et al., 1986). Third, repeated measurement and temporal separation extend cross-sectional evidence by showing prospective panel associations after accounting for prior levels (Cole and Maxwell, 2003). These contributions are best viewed as a refinement and integration of existing theory rather than a wholly new causal model, particularly because the conventional CLPM does not isolate within-person dynamics (Hamaker et al., 2015; Mulder and Hamaker, 2021).

The practical implications should be considered in light of the modest effect sizes and observational design. Accordingly, the following suggestions should be treated as testable program-design hypotheses rather than as established mental-health intervention recommendations. University physical education programs may consider supporting all three adherence components: explaining the rationale and structure of the prescription, helping students implement appropriate frequency, intensity, duration, progression, and technique, and teaching planning, self-monitoring, and adjustment strategies (Garber et al., 2011; He et al., 2025). Instructors may also draw attention to breathing, bodily tension, attentional focus, and emotional responses during practice (Wayne and Kaptchuk, 2008; Qi et al., 2022; Gross, 1998) and connect these experiences with stress-management education (Cohen et al., 1983; VanKim and Nelson, 2013; Sheng et al., 2024). These candidate approaches should complement, rather than replace, professional mental health services for students with clinically significant anxiety. Before they are recommended as strategies for anxiety prevention or reduction, randomized or well-controlled quasi-experimental studies should test whether adherence-focused course modifications increase adherence and whether experimentally induced differences are followed by meaningful changes in emotion regulation difficulties, perceived stress, and anxiety symptoms.

### 4.7 Limitations and future directions

Several limitations should be acknowledged. First, although the three-wave design establishes temporal separation and strengthens prospective inference, the study remains observational. The coefficients may reflect unmeasured confounding, selection processes, or stable individual differences; consequently, the results should be described as longitudinal associations rather than causal effects (Cole and Maxwell, 2003; Maxwell and Cole, 2007). Randomized or quasi-experimental studies are required to determine whether interventions that improve multidimensional adherence are followed by changes in emotion regulation difficulties, perceived stress, and anxiety symptoms (Du et al., 2022; Lin et al., 2022; Qi et al., 2022). The four- and five-month intervals may also miss shorter-term fluctuations and should not be assumed to represent the optimal temporal lag for these processes.

Second, all core constructs were assessed by self-report. Harman’s single-factor test and full-collinearity VIFs did not indicate a dominant single factor or severe full collinearity, but these diagnostics are not sufficiently sensitive to rule out common method variance (Podsakoff et al., 2003; Podsakoff et al., 2012; Kock, 2015). Shared response tendencies, social desirability, and recall bias may therefore have inflated some associations. Future studies should combine self-reports with attendance and practice logs, wearable activity indicators, instructor ratings, ecological momentary assessment, physiological stress markers, or appropriately specified latent method-factor or marker-variable approaches.

Third, recruitment from two universities in Tianjin limits generalizability despite the inclusion of university, academic field, residence, and socioeconomic status as covariates. Replication across regions, university types, and cultural contexts is needed. Fourth, traditional Chinese ethnic sports were treated as a broad category, although Tai Chi, Baduanjin, Wushu, shuttlecock kicking, and dragon dance differ in intensity, social interaction, attentional demands, and cultural meaning (Wayne and Kaptchuk, 2008; Qi et al., 2022). Activity-specific and dimension-specific adherence analyses may reveal heterogeneous associations that are obscured by the overall composite.

Finally, the traditional CLPM does not separate stable between-person differences from within-person deviations, so its cross-lagged coefficients should not be interpreted as intraindividual processes (Hamaker et al., 2015; Mulder and Hamaker, 2021). More specifically, conventional CLPM estimates do not decompose observed scores into stable person-specific components and time-varying within-person deviations; the cross-lagged paths may therefore reflect a mixture of enduring between-person differences and within-person temporal covariation. This distinction is especially important given the substantial autoregressive stability observed in the present study. Accordingly, the model cannot determine whether a student who becomes more adherent than their own typical level subsequently experiences lower-than-usual emotion regulation difficulties, perceived stress, or anxiety, and temporal precedence in the CLPM should not be treated as evidence of an intraindividual explanatory mechanism. Future work should compare CLPM and RI-CLPM estimates and, where sample size and measurement structure permit, use random-intercept, multilevel, or latent growth approaches. In addition, the focal mediation analysis used the prespecified T1 adherence → T2 mediators → T3 anxiety contrast with baseline controls rather than a fully dynamic repeated-measures mediation model. Autoregressive mediation models incorporating all wave-specific indirect paths could clarify whether the observed associations replicate across intervals and whether within-person changes in adherence precede within-person changes in the proposed mediators and anxiety (Cole and Maxwell, 2003; Maxwell and Cole, 2007).

## 5 Conclusion

This three-wave longitudinal study examined the associations among adherence to traditional Chinese ethnic sports, emotion regulation difficulties, perceived stress, and anxiety symptoms among college students. The findings showed that adherence to traditional Chinese ethnic sports was prospectively and inversely associated with subsequent emotion regulation difficulties and perceived stress, and that both psychological variables were prospectively and positively associated with later anxiety symptoms. The longitudinal parallel mediation model further showed that emotion regulation difficulties and perceived stress showed two significant indirect associations linking adherence to traditional Chinese ethnic sports with anxiety symptoms.

Overall, the findings suggest that the mental-health relevance of traditional Chinese ethnic sports may depend not only on participation but also on the multidimensional quality and continuity with which prescribed practice is implemented. Two modest parallel indirect associations were observed between higher adherence and lower later anxiety symptoms, involving lower subsequent emotion regulation difficulties and perceived stress. These model-based indirect associations describe temporally ordered covariance in observational data and should not be interpreted as evidence that adherence improves emotion regulation, reduces perceived stress, or produces anxiety reduction. The study therefore offers a refined, implementation-focused account of existing physical activity–mental health theory, while the modest effect sizes, self-report measurement, conventional CLPM, and observational design require cautious interpretation. Experimental and within-person longitudinal studies are needed before adherence can be regarded as a causal intervention target.

## Data Availability

The original contributions presented in the study are included in the article/Supplementary material, further inquiries can be directed to the corresponding author.
